# Supplementary material for: A Review of Termite Species and Their Distribution in Thailand
Source: Insects. 2022 Feb 10;13(2):186. doi: 10.3390/insects13020186 (PMC8879794; doi:10.3390/insects13020186)
Supplement: Supplementary file 1 [file insects-13-00186-s001.zip › insects-1554048-supplementary.pdf]

Supplementary Table s 1: Locations of termite species previously published in publications in Thailand between 1965 and 2021

Province	District	Geographic Location1/	Termite Species	Citation
**Northern region**				
Chiang Mai	Mueang Chiang Mai	18.7985328°N, 98.9346081°E	*Schedorhinotermes sarawakensis*	[20]
		18.7985328°N, 98.9346081°E	*Macrotermes annandalei*	[20]
		18.7985328°N, 98.9346081°E	*Hypotermes xenotermitis*	[20]
		18.7985328°N, 98.9346081°E	*Microtermes pakistanicus*	[20]
		18.7985328°N, 98.9346081°E	*Microtermes obesi*	[20]
		18.7985328°N, 98.9346081°E	*Odontotermes proformosanus*	[20]
		18.7985328°N, 98.9346081°E	*Odontotermes formosanus*	[20]
		18.7985328°N, 98.9346081°E	*Odontotermes feae*	[20]
		18.7985328°N, 98.9346081°E	*Globitermes sulphureus*	[20]
		18.7985328°N, 98.9346081°E	*Microcerotermes crassus*	[20]
		18.7985328°N, 98.9346081°E	*Microcerotermes minutus*	[20]
		18.7985328°N, 98.9346081°E	*Dicuspiditermes garthwaitei*	[20]
		18.7985328°N, 98.9346081°E	*Mirocapritermes concaveus*	[20]
		18.7985328°N, 98.9346081°E	*Speculitermes rongrensis*	[20]
		18.7985328°N, 98.9346081°E	*Speculitermes* sp. A	[20]
		18.7985328°N, 98.9346081°E	*Lacessititermes* sp. A	[20]
**Central region**				

Province	District	Geographic Location[1/]	Termite Species	Citation
Saraburi	Muaek Lek	14.6500000°N, 101.2000000°E	*Odontotermes formosanus*	[18]
		14.6500000°N, 101.2000000°E	*Odontotermes paraoblongatus*	[18]
		14.6500000°N, 101.2000000°E	*Hypotermes makhamensis*	[18]
		14.6500000°N, 101.2000000°E	*Microtermes pakistanicus*	[18]
		14.6500000°N, 101.2000000°E	*Microcerotermes crassus*	[18]
Pitsanulok	Nakhon Thai	16.8333333°N, 100.9000000°E	*Glyptotermes pinangae*	[18]
		16.8333333°N, 100.9000000°E	*Coptotermes havilandi*	[18]
		16.8333333°N, 100.9000000°E	*Schedorhinotermes medioobscurus*	[18]
		16.8333333°N, 100.9000000°E	*Macrotermes gilvus*	[18]
		16.8333333°N, 100.9000000°E	*Odontotermes longignathus*	[18]
		16.8333333°N, 100.9000000°E	*Microtermes obesi*	[18]
		16.8333333°N, 100.9000000°E	*Microtermes pakistanicus*	[18]
		16.8333333°N, 100.9000000°E	*Indotermes thailandis*	[18]
		16.8333333°N, 100.9000000°E	*Speculitermes macrodentatus*	[18]
		16.8333333°N, 100.9000000°E	*Microcerotermes crassus*	[18]
		16.8333333°N, 100.9000000°E	*Globitermes sulphureus*	[18]
		16.8333333°N, 100.9000000°E	*Nasutitermes matangensiformis*	[18]
		16.8333333°N, 100.9000000°E	*Nasutitermes tungsalangensis*	[18]
		16.8333333°N, 100.9000000°E	*Bulbitermes laticephalus*	[18]
		16.8333333°N, 100.9000000°E	*Hospitalitermes ataramensis*	[18]

Province	District	Geographic Location1/	Termite Species	Citation
		16.8333333°N, 100.9000000°E	*Termes cosmis*	[18]
		16.8333333°N, 100.9000000°E	*Termes propinquus*	[18]
		16.8333333°N, 100.9000000°E	*Mirocapritermes concaveus*	[18]
		16.8333333°N, 100.9000000°E	*Pericapritermes latignathus*	[18]
		16.8333333°N, 100.9000000°E	*Pericapritermes semarangi*	[18]
		16.8333333°N, 100.9000000°E	*Dicuspiditermes garthwaitei*	[18]
		16.8333333°N, 100.9000000°E	*Procapritermes longignathus*	[18]
		16.8333333°N, 100.9000000°E	*Procapritermes parasilvaticus*	[18]
Pitsanulok	Wang	16.8500000°N, 100.5166667°E	*Prorhinotermes tibiaoensiformis*	[18]
	Nok An	16.8500000°N, 100.5166667°E	*Microcerotermes minutus*	[18]
		16.8500000°N, 100.5166667°E	*Nasutitermes perparvus*	[18]
		16.8500000°N, 100.5166667°E	*Termes cosmis*	[18]
		16.8500000°N, 100.5166667°E	*Termes propinquus*	[18]
**Northeastern region**				
Khon Kaen	Ban Fang	16.3800000°N, 102.6200000°E	*Odontotermes formosanus*	[25]
		16.3800000°N, 102.6200000°E	*Hospitalitermes ataramensis*	[25]
		16.3800000°N, 102.6200000°E	*Angulitermes sp.*	[25]
		16.3800000°N, 102.6200000°E	*Microcerotermes sp.*	[25]
		16.3800000°N, 102.6200000°E	*Pericapritermes sp*	[25]
Nakhon	Pak	14.6333333°N, 101.2666667°E	*Cryptotermes thailandis*	[18]

Province	District	Geographic Location[1/]	Termite Species	Citation
Ratchasima	Chong	14.6333333°N, 101.2666667°E	*Microcerotermes crassus*	[18]
Nakhon	Wang	14.5000000°N, 101.9333333°E	*Coptotermes curvignathus*	[24]
Ratchasima	Nam	14.5000000°N, 101.9333333°E	*Schedorhinotermes medioobscurus*	[24]
	Khiao	14.5000000°N, 101.9333333°E	*Schedorhinotermes* sp.	[24]
		14.5000000°N, 101.9333333°E	*Microcerotermes crassus*	[24]
		14.5000000°N, 101.9333333°E	*Globitermes sulphureus*	[24]
		14.5000000°N, 101.9333333°E	*Dicuspiditermes garthwaitei*	[24]
		14.5000000°N, 101.9333333°E	*Dicuspiditermes makhamensis*	[24]
		14.5000000°N, 101.9333333°E	*Mirocapritermes concaveus*	[24]
		14.5000000°N, 101.9333333°E	*Mirocapritermes* sp.1	[24]
		14.5000000°N, 101.9333333°E	*Pericapritermes semarangi*	[24]
		14.5000000°N, 101.9333333°E	*Procapritermes prosetiger*	[24]
		14.5000000°N, 101.9333333°E	*Termes propinquus*	[24]
		14.5000000°N, 101.9333333°E	*Bulbitermes makhamensis*	[24]
		14.5000000°N, 101.9333333°E	*Hospitalitermes bicolor*	[24]
		14.5000000°N, 101.9333333°E	*Hypotermes makhamensis*	[24]
		14.5000000°N, 101.9333333°E	*Hypotermes* sp.1	[24]
		14.5000000°N, 101.9333333°E	*Macrotermes annandalei*	[24]
		14.5000000°N, 101.9333333°E	*Macrotermes gilvus*	[24]
		14.5000000°N, 101.9333333°E	*Microtermes obesi*	[24]

Province	District	Geographic Location[1/]	Termite Species	Citation
		14.5000000°N, 101.9333333°E	*Microtermes pakistanicus*	[24]
		14.5000000°N, 101.9333333°E	*Odontotermes feae*	[24]
		14.5000000°N, 101.9333333°E	*Odontotermes maesodensis*	[24]
		14.5000000°N, 101.9333333°E	*Euhamitermes* sp. 1	[24]
		14.5000000°N, 101.9333333°E	*Spculitermes* sp. 1	[24]
		14.5000000°N, 101.9333333°E	Termitinae spp.[3/]	[24]
		14.5000000°N, 101.9333333°E	Macrotermitinae spp.1[3/]	[24]
		14.5000000°N, 101.9333333°E	Apicotermitinae sp.2[3/]	[24]
		14.5000000°N, 101.9333333°E	Termitidae sp.3[3/]	[24]
Nakhon Ratchasima	Khao Yai	14.4333333°N, 101.0333333°E	*Nasutitermes matangensiformis*	[18]
		14.4333333°N, 101.0333333°E	*Nasutitermes tungsalangensis*	[18]
		14.4333333°N, 101.0333333°E	*Bulbitermes laticephalus*	[18]
		14.4333333°N, 101.0333333°E	*Aciculitermes maymyoensis*	[18]
		14.4333333°N, 101.0333333°E	*Mirocapritermes concaveus*	[18]
		14.4333333°N, 101.0333333°E	*Procapritermes prosetiger*	[18]
Nakhon Ratchasima	Wang Nam Khiao	14.4938800°N, 101.8749400°E	*Macrotermes annandalei*	[26]
		14.4939600°N, 101.8751300°E	*Macrotermes annandalei*	[26]
		14.4936600°N, 101.8766400°E	*Ancistrotermes pakistanicus*	[26]
		14.4940900°N, 101.8762300°E	*Odontotermes feae*	[26]
Chaiyaphum	Khon San	16.5287800°N, 101.6601800°E	*Ancistrotermes* sp.	[26]

Province	District	Geographic Location[1/]	Termite Species	Citatio n
		16.5287800°N, 101.6601800°E	*Ancistrotermes* sp.	[26]
**Eastern region**				
Chanthaburi	Mueang Chanthaburi	12.6000000°N, 102.1166667°E	*Coptotermes curvignathus*	[18]
		12.6000000°N, 102.1166667°E	*Schedorhinotermes medioobscurus*	[18]
		12.6000000°N, 102.1166667°E	*Schedorhinotermes rectangularis*	[18]
		12.6000000°N, 102.1166667°E	*Macrotermes carbonarius*	[18]
		12.6000000°N, 102.1166667°E	*Microtermes pakistanicus*	[18]
		12.6000000°N, 102.1166667°E	*Microcerotermes crassus*	[18]
		12.6000000°N, 102.1166667°E	*Globitermes sulphureus*	[18]
		12.6000000°N, 102.1166667°E	*Amitermes longignathus*	[18]
		12.6000000°N, 102.1166667°E	*Nasutitermes johoricus*	[18]
		12.6000000°N, 102.1166667°E	*Termes cosmis*	[18]
		12.6000000°N, 102.1166667°E	*Dicuspiditermes makhamensis*	[18]
Chanthaburi	Khao Khitchakut	12.8383333°N, 102.1533333°E	*Hospitalitermes sp.*	[21]
		12.8383333°N, 102.1533333°E	*Bulbitermes sp.*	[21]
Chanthaburi	Khao Khitchakut	12.8166667°N, 102.1500000°E	*Schedorhinotermes medioobscurus*	[23]
		12.8166667°N, 102.1500000°E	*Schedorhinotermes rectangularis*	[23]
		12.8166667°N, 102.1500000°E	*Ancistrotermes pakistanicus*	[23]
		12.8166667°N, 102.1500000°E	*Hypotermes makhamensis*	[23]
		12.8166667°N, 102.1500000°E	*Macrotermes annandalei*	[23]

Province	District	Geographic Location[1/]	Termite Species	Citation
		12.8166667°N, 102.1500000°E	*Microtermes obesi*	[23]
		12.8166667°N, 102.1500000°E	*Odontotermes feae*	[23]
		12.8166667°N, 102.1500000°E	*Odontotermes proformosanus*	[23]
		12.8166667°N, 102.1500000°E	*Bulbitermes laticephalus*	[23]
		12.8166667°N, 102.1500000°E	*Bulbitermes parapusillus*	[23]
		12.8166667°N, 102.1500000°E	*Bulbitermes prabhae*	[23]
		12.8166667°N, 102.1500000°E	*Bulbitermes* sp.1	[23]
		12.8166667°N, 102.1500000°E	*Lacessititermes* sp.1	[23]
		12.8166667°N, 102.1500000°E	*Nasutitermes johoricus*	[23]
		12.8166667°N, 102.1500000°E	*Nasutitermes matangensis*	[23]
		12.8166667°N, 102.1500000°E	*Nasutitermes* sp.1	[23]
		12.8166667°N, 102.1500000°E	*Nasutitermes* sp.2	[23]
		12.8166667°N, 102.1500000°E	*Euhamitermes* sp.1	[23]
		12.8166667°N, 102.1500000°E	*Euhamitermes* sp.2	[23]
		12.8166667°N, 102.1500000°E	*Dicuspiditermes spinitibialis*	[23]
		12.8166667°N, 102.1500000°E	*Globitermes* sp.1	[23]
		12.8166667°N, 102.1500000°E	*Microcerotermes crassus*	[23]
		12.8166667°N, 102.1500000°E	*Mirocapritermes concaveus*	[23]
		12.8166667°N, 102.1500000°E	*Mirocapritermes* sp.1	[23]
		12.8166667°N, 102.1500000°E	*Pericapritermes latignathus*	[23]

Province	District	Geographic Location[1/]	Termite Species	Citation
		12.8166667°N, 102.1500000°E	*Pericapritermes semarangi*	[23]
		12.8166667°N, 102.1500000°E	*Procapritermes parasilvaticus*	[23]
		12.8166667°N, 102.1500000°E	*Procapritermes prosetiger*	[23]
		12.8166667°N, 102.1500000°E	*Termes cosmis*	[23]
		12.8166667°N, 102.1500000°E	Kalotermitidae sp.1	[23]
Chanthaburi	Makham	12.6666667°N, 102.2000000°E	*Schedorhinotermes medioobscurus*	[18]
		12.6666667°N, 102.2000000°E	*Macrotermes gilvus*	[18]
		12.6666667°N, 102.2000000°E	*Odontotermes feae*	[18]
		12.6666667°N, 102.2000000°E	*Hypotermes makhamensis*	[18]
		12.6666667°N, 102.2000000°E	*Bulbitermes prabhae*	[18]
		12.6666667°N, 102.2000000°E	*Bulbitermes makhamensis*	[18]
		12.6666667°N, 102.2000000°E	*Termes huayangensis*	[18]
		12.6666667°N, 102.2000000°E	*Termes cosmis*	[18]
		12.6666667°N, 102.2000000°E	*Pericapritermes semarangi*	[18]
		12.6666667°N, 102.2000000°E	*Dicuspiditermes makhamensis*	[18]
Chanthaburi	Prew	12.5166667°N, 102.1833333°E	*Glyptotermes pinangae*	[18]
		12.5166667°N, 102.1833333°E	*Coptotermes curvignathus*	[18]
		12.5166667°N, 102.1833333°E	*Macrotermes carbonarius*	[18]
		12.5166667°N, 102.1833333°E	*Microcerotermes crassus*	[18]
		12.5166667°N, 102.1833333°E	*Nasutitermes matangensiformis*	[18]

Province	District	Geographic Location1/	Termite Species	Citation
		12.5166667°N, 102.1833333°E	*Nasutitermes johoricus*	[18]
		12.5166667°N, 102.1833333°E	*Bulbitermes parapusillus*	[18]
		12.5166667°N, 102.1833333°E	*Mirocapritermes prewensis*	[18]
**Western region**				
Prachuap Khiri Khan	Thap Sakae	11.5833333°N, 99.6333333°E	*Coptotermes havilandi* (Now as *C. gestroi*)	[18]
		11.5833333°N, 99.6333333°E	*Macrotermes gilvus*	[18]
		11.5833333°N, 99.6333333°E	*Macrotermes annandalei*	[18]
		11.5833333°N, 99.6333333°E	*Odontotermes proformosanus*	[18]
		11.5833333°N, 99.6333333°E	*Microtermes obesi*	[18]
		11.5833333°N, 99.6333333°E	*Microcerotermes crassus*	[18]
		11.5833333°N, 99.6333333°E	*Globitermes sulphureus*	[18]
		11.5833333°N, 99.6333333°E	*Amitermes longignathus*	[18]
		11.5833333°N, 99.6333333°E	*Amitermes dentatus*	[18]
		11.5833333°N, 99.6333333°E	*Nasutitermes perparvus*	[18]
		11.5833333°N, 99.6333333°E	*Bulbitermes prabhae*	[18]
		11.5833333°N, 99.6333333°E	*Termes huayangensis*	[18]
		11.5833333°N, 99.6333333°E	*Termes cosmis*	[18]
		11.5833333°N, 99.6333333°E	*Termes propinquus*	[18]
Tak	Mueang	16.7833333°N, 99.1500000°E	*Macrotermes gilvus*	[18]

Province	District	Geographic Location1/	Termite Species	Citation
	Tak	16.7833333°N, 99.1500000°E	*Odontotermes takensis*	[18]
		16.7833333°N, 99.1500000°E	*Microcerotermes crassus*	[18]
		16.7833333°N, 99.1500000°E	*Globitermes sulphureus*	[18]
		16.7833333°N, 99.1500000°E	*Termes propinquus*	[18]
**Southern region**				
Trang	Na Yong	7.5500000°N, 99.7833333°E	*Glyptotermes brevicaudatus*	[18]
		7.5500000°N, 99.7833333°E	*Glyptotermes kachongensis*	[18]
		7.5500000°N, 99.7833333°E	*Coptotermes premrasmii*	[18]
		7.5500000°N, 99.7833333°E	*Coptotermes havilandi* (Now as *C. gestroi*)	[18]
		7.5500000°N, 99.7833333°E	*Coptotermes curvignathus*	[18]
		7.5500000°N, 99.7833333°E	*Coptotermes kalshoveni*	[18]
		7.5500000°N, 99.7833333°E	*Schedorhinotermes medioobscurus*	[18]
		7.5500000°N, 99.7833333°E	*Schedorhinotermes sarawakensis*	[18]
		7.5500000°N, 99.7833333°E	*Macrotermes gilvus*	[18]
		7.5500000°N, 99.7833333°E	*Macrotermes carbonarius*	[18]
		7.5500000°N, 99.7833333°E	*Macrotermes malaccensis*	[18]
		7.5500000°N, 99.7833333°E	*Odontotermes proformosanus*	[18]
		7.5500000°N, 99.7833333°E	*Odontotermes oblongathus*	[18]
		7.5500000°N, 99.7833333°E	*Odontotermes feae*	[18]

Province	District	Geographic Location1/	Termite Species	Citation
		7.5500000°N, 99.7833333°E	*Odontotermes longignathus*	[18]
		7.5500000°N, 99.7833333°E	*Hypotermes xenotermitis*	[18]
		7.5500000°N, 99.7833333°E	*Microtermes obesi*	[18]
		7.5500000°N, 99.7833333°E	*Microtermes pakistanicus*	[18]
		7.5500000°N, 99.7833333°E	*Euhamitermes hamatus*	[18]
		7.5500000°N, 99.7833333°E	*Microcerotermes paracelebensis*	[18]
		7.5500000°N, 99.7833333°E	*Microcerotermes crassus*	[18]
		7.5500000°N, 99.7833333°E	*Globitermes sulphureus*	[18]
		7.5500000°N, 99.7833333°E	*Amitermes dentatus*	[18]
		7.5500000°N, 99.7833333°E	*Havilanditermes proatripennis*	[18]
		7.5500000°N, 99.7833333°E	*Nasutitermes matangensiformis*	[18]
		7.5500000°N, 99.7833333°E	*Nasutitermes fuscipennis*	[18]
		7.5500000°N, 99.7833333°E	*Nasutitermes johoricus*	[18]
		7.5500000°N, 99.7833333°E	*Hospitalitermes jepsoni*	[18]
		7.5500000°N, 99.7833333°E	*Termes propinquus*	[18]
		7.5500000°N, 99.7833333°E	*Mirocapritermes connectens*	[18]
		7.5500000°N, 99.7833333°E	*Mirocapritermes latignathus*	[18]
		7.5500000°N, 99.7833333°E	*Pericapritermes latignathus*	[18]
		7.5500000°N, 99.7833333°E	*Procapritermes prosetiger*	[18]
Trang	Kan	7.3833333°N, 99.5000000°E	*Schedorhinotermes medioobscurus*	[18]

Province	District	Geographic Location[1/]	Termite Species	Citation
	Tang	7.3833333°N, 99.5000000°E	*Macrotermes carbonarius*	[18]
		7.3833333°N, 99.5000000°E	*Microtermes pakistanicus*	[18]
		7.3833333°N, 99.5000000°E	*Microcerotermes crassus*	[18]
		7.3833333°N, 99.5000000°E	*Globitermes sulphureus*	[18]
		7.3833333°N, 99.5000000°E	*Nasutitermes dimorphus*	[18]
		7.3833333°N, 99.5000000°E	*Nasutitermes johoricus*	[18]
Songkhla	Sadao	6.5750000°N, 100.4008833°E	*Microcerotermes serrula*	[22]

[1/] Geographic Location (Latitude and Longitude) was recorded by Google Earth version 9.121.0.5 and Google Maps application version 10.61.2.

[2/] Unknown species in each subfamily and family

Supplementary Table S2: Locations of termite species previously published in annual reports and theses in Thailand between 1965 and 2021

Province	District	Geographic Location[1/]	Termite Species	Citation
**Northern region**				
Chiang Mai	Hot	18.2225625°N, 98.4818299°E	*Coptotermes gestroi*	[30]
		18.2225625°N, 98.4818299°E	*Coptotermes* sp.1	[30]
		18.2225625°N, 98.4818299°E	*Macrotermes annandalei*	[30]
		18.2225625°N, 98.4818299°E	*Macrotermes gilvus*	[30]
		18.2225625°N, 98.4818299°E	*Microtermes obesi*	[30]
		18.2225625°N, 98.4818299°E	*Hypotermes makhamensis*	[30]
		18.2225625°N, 98.4818299°E	*Odontotermes takensis*	[30]
		18.2225625°N, 98.4818299°E	*Odontotermes* sp.2	[30]
		18.2225625°N, 98.4818299°E	*Odontotermes* sp.16	[30]
		18.2225625°N, 98.4818299°E	*Microcerotermes crassus*	[30]
		18.2225625°N, 98.4818299°E	*Globitermes sulphureus*	[30]
		18.2225625°N, 98.4818299°E	*Termes cosmis*	[30]
		18.2225625°N, 98.4818299°E	*Hospitalitermes ataramensis*	[30]

Province	District	Geographic Location[1/]	Termite Species	Citation
Chiang Mai	Mueang Chiang Mai	18.7756009°N, 98.8974995°E	*Schedorhinotermes medioobscurus*	[30]
		18.7756009°N, 98.8974995°E	*Coptotermes gestroi*	[30]
		18.7756009°N, 98.8974995°E	*Coptotermes curvignathus*	[30]
		18.7756009°N, 98.8974995°E	*Macrotermes annandalei*	[30]
		18.7756009°N, 98.8974995°E	*Macrotermes maesodensis*	[30]
		18.7756009°N, 98.8974995°E	*Microtermes obesi*	[30]
		18.7756009°N, 98.8974995°E	*Ancistrotermes pakestanicus*	[30]
		18.7756009°N, 98.8974995°E	*Hypotermes makhamensis*	[30]
		18.7756009°N, 98.8974995°E	*Odontotermes proformosanus*	[30]
		18.7756009°N, 98.8974995°E	*Odontotermes takensis*	[30]
		18.7756009°N, 98.8974995°E	*Odontotermes* sp.2	[30]
		18.7756009°N, 98.8974995°E	*Odontotermes* sp.9	[30]
		18.7756009°N, 98.8974995°E	*Microcerotermes crassus*	[30]
		18.7756009°N, 98.8974995°E	*Globitermes sulphureus*	[30]
		18.7756009°N, 98.8974995°E	*Euhamitermes hamatus*	[30]
		18.7756009°N, 98.8974995°E	*Speculitermes* sp.	[30]
		18.7756009°N, 98.8974995°E	*Termes cosmis*	[30]
		18.7756009°N, 98.8974995°E	*Dicuspiditermes garthwaitei*	[30]
		18.7756009°N, 98.8974995°E	*Mirocapritermes latignathus*	[30]
		18.7756009°N, 98.8974995°E	*Pericapritermes semarangi*	[30]

Province	District	Geographic Location[1/]	Termite Species	Citation
		18.7756009°N, 98.8974995°E	*Procapritermes parasilvaticus*	[30]
		18.7756009°N, 98.8974995°E	*Procapritermes* sp.1	[30]
		18.7756009°N, 98.8974995°E	*Nasutitermes fuscipennis*	[30]
		18.7756009°N, 98.8974995°E	*Bulbitermes prabhae*	[30]
		18.7756009°N, 98.8974995°E	*Bulbitermes laticephalus*	[30]
		18.7756009°N, 98.8974995°E	*Bulbitermes* sp.1	[30]
		18.7756009°N, 98.8974995°E	*Hospitalitermes ataramensis*	[30]
Chiang Mai	Doi Saket	18.9663333°N, 99.2420833°E	*Coptotermes gestroi*	[30]
		18.9663333°N, 99.2420833°E	*Coptotermes* sp.1	[30]
		18.9663333°N, 99.2420833°E	*Macrotermes annandalei*	[30]
		18.9663333°N, 99.2420833°E	*Macrotermes gilvus*	[30]
		18.9663333°N, 99.2420833°E	*Ancistrotermes pakestanicus*	[30]
		18.9663333°N, 99.2420833°E	*Hypotermes makhamensis*	[30]
		18.9663333°N, 99.2420833°E	*Odontotermes proformosanus*	[30]
		18.9663333°N, 99.2420833°E	*Odontotermes takensis*	[30]
		18.9663333°N, 99.2420833°E	*Microcerotermes crassus*	[30]
		18.9663333°N, 99.2420833°E	*Globitermes sulphureus*	[30]
		18.9663333°N, 99.2420833°E	*Termes cosmis*	[30]
		18.9663333°N, 99.2420833°E	*Dicuspiditermes garthwaitei*	[30]
		18.9663333°N, 99.2420833°E	*Pericapritermes* sp.D	[30]

Province	District	Geographic Location1/	Termite Species	Citation
		18.9663333°N, 99.2420833°E	*Nasutitermes matangensiformis*	[30]
		18.9663333°N, 99.2420833°E	*Bulbitermes prabhae*	[30]
		18.9663333°N, 99.2420833°E	*Hospitalitermes jepsoni*	[30]
Chiang Mai	Chom Thong	18.5700442°N, 98.5153422°E	*Macrotermes maesodensis*	[30]
		18.5700442°N, 98.5153422°E	*Odontotermes proformosanus*	[30]
		18.5700442°N, 98.5153422°E	*Odontotermes formosanus*	[30]
		18.5700442°N, 98.5153422°E	*Microcerotermes crassus*	[30]
		18.5700442°N, 98.5153422°E	*Microcerotermes paracelebensis*	[30]
		18.5700442°N, 98.5153422°E	*Dicuspiditermes garthwaitei*	[30]
		18.5700442°N, 98.5153422°E	*Pericapritermes semarangi*	[30]
		18.5700442°N, 98.5153422°E	*Pericapritermes* sp.E	[30]
		18.5700442°N, 98.5153422°E	*Bulbitermes prabhae*	[30]
		18.5700442°N, 98.5153422°E	*Bulbitermes* sp.1	[30]
Lampang	Mueang Pan	18.8368989°N, 99.4713105°E	*Schedorhinotermes medioobscurus*	[30]
		18.8368989°N, 99.4713105°E	*Schedorhinotermes sarawakensis*	[30]
		18.8368989°N, 99.4713105°E	*Coptotermes gestroi*	[30]
		18.8368989°N, 99.4713105°E	*Coptotermes* sp.1	[30]
		18.8368989°N, 99.4713105°E	*Macrotermes annandalei*	[30]
		18.8368989°N, 99.4713105°E	*Macrotermes gilvus*	[30]
		18.8368989°N, 99.4713105°E	*Microtermes obesi*	[30]

Province	District	Geographic Location[1/]	Termite Species	Citation
		18.8368989°N, 99.4713105°E	*Ancistrotermes pakestanicus*	[30]
		18.8368989°N, 99.4713105°E	*Hypotermes makhamensis*	[30]
		18.8368989°N, 99.4713105°E	*Odontotermes proformosanus*	[30]
		18.8368989°N, 99.4713105°E	*Odontotermes longignathus*	[30]
		18.8368989°N, 99.4713105°E	*Odontotermes takensis*	[30]
		18.8368989°N, 99.4713105°E	*Odontotermes* sp.1	[30]
		18.8368989°N, 99.4713105°E	*Odontotermes* sp.2	[30]
		18.8368989°N, 99.4713105°E	*Odontotermes* sp.3	[30]
		18.8368989°N, 99.4713105°E	*Odontotermes* sp.7	[30]
		18.8368989°N, 99.4713105°E	*Odontotermes* sp.8	[30]
		18.8368989°N, 99.4713105°E	*Odontotermes* sp.12	[30]
		18.8368989°N, 99.4713105°E	*Microcerotermes crassus*	[30]
		18.8368989°N, 99.4713105°E	*Globitermes sulphureus*	[30]
		18.8368989°N, 99.4713105°E	*Euhamitermes* sp.	[30]
		18.8368989°N, 99.4713105°E	*Speculitermes* sp.	[30]
		18.8368989°N, 99.4713105°E	*Termes cosmis*	[30]
		18.8368989°N, 99.4713105°E	*Termes propinquus*	[30]
		18.8368989°N, 99.4713105°E	*Dicuspiditermes garthwaitei*	[30]
		18.8368989°N, 99.4713105°E	*Dicuspiditermes* sp.1	[30]
		18.8368989°N, 99.4713105°E	*Mirocapritermes latignathus*	[30]

Province	District	Geographic Location[1/]	Termite Species	Citation
		18.8368989°N, 99.4713105°E	*Mirocapritermes concaveus*	[30]
		18.8368989°N, 99.4713105°E	*Mirocapritermes* sp.1	[30]
		18.8368989°N, 99.4713105°E	*Procapritermes parasilvaticus*	[30]
		18.8368989°N, 99.4713105°E	*Procapritermes* sp1	[30]
		18.8368989°N, 99.4713105°E	*Nasutitermes matangensiformis*	[30]
		18.8368989°N, 99.4713105°E	*Bulbitermes prabhae*	[30]
		18.8368989°N, 99.4713105°E	*Hospitalitermes jepsoni*	[30]
		18.8368989°N, 99.4713105°E	*Hospitalitermes* sp.2	[30]
**Central region**				
Patumthani	Khlong	14.0366751°N, 100.7276619°E	*Coptotermes* sp.	[35]
	Luang	14.0366751°N, 100.7276619°E	*Parrhinotermes* sp.	[35]
		14.0366751°N, 100.7276619°E	*Macrotermes* sp.	[35]
		14.0366751°N, 100.7276619°E	*Odontotermes* sp.	[35]
		14.0366751°N, 100.7276619°E	*Microcerotermes* sp.	[35]
		14.0366751°N, 100.7276619°E	*Termes* sp.	[35]
**Northeastern region**				
Nakhon	Wang	14.5000000°N, 101.9166667°E	*Glyptotermes brevicaudatus*	[36]
Ratchasima	Nam	14.5000000°N, 101.9166667°E	*Schedorhinotermes* sp.	[36]
	Khiao	14.5000000°N, 101.9166667°E	*Coptotermes curvignathus*	[36]
		14.5000000°N, 101.9166667°E	*Macrotermes carbonarius*	[36]

Province	District	Geographic Location[1/]	Termite Species	Citation
		14.5000000°N, 101.9166667°E	*Macrotermes gilvus*	[36]
		14.5000000°N, 101.9166667°E	*Macrotermes annandalie*	[36]
		14.5000000°N, 101.9166667°E	*Odontotermes longignathus*	[36]
		14.5000000°N, 101.9166667°E	*Odontotermes feae*	[36]
		14.5000000°N, 101.9166667°E	*Odontotermes proformosanus*	[36]
		14.5000000°N, 101.9166667°E	*Hypotermes makhamensis*	[36]
		14.5000000°N, 101.9166667°E	*Microtermes* sp.	[36]
		14.5000000°N, 101.9166667°E	*Ancistrotermes pakistanicus*	[36]
		14.5000000°N, 101.9166667°E	*Amitermes* sp.	[36]
		14.5000000°N, 101.9166667°E	*Globitermes sulphureus*	[36]
		14.5000000°N, 101.9166667°E	*Microcerotermes crassus*	[36]
		14.5000000°N, 101.9166667°E	*Dicuspiditermes makhamensis*	[36]
		14.5000000°N, 101.9166667°E	*Mirocapritermes* sp.	[36]
		14.5000000°N, 101.9166667°E	*Homallotermes* sp.	[36]
		14.5000000°N, 101.9166667°E	*Procapritermes* sp.	[36]
		14.5000000°N, 101.9166667°E	*Pericapritermes* sp.	[36]
		14.5000000°N, 101.9166667°E	*Termes* sp.	[36]
		14.5000000°N, 101.9166667°E	*Nasutitermes* sp.	[36]
		14.5000000°N, 101.9166667°E	Kalotermitinae sp.1[3/]	[36]
		14.5000000°N, 101.9166667°E	Nasutitermitinae sp.1[3/]	[36]

Province	District	Geographic Location[1/]	Termite Species	Citation
		14.5000000°N, 101.9166667°E	Nasutitermitinae sp.2[3/]	[36]
Khon Kaen	Mueang Khon Kaen	16.4700000°N,102.8200000°E	*Macrotermes gilvus*	[27]
		16.4700000°N,102.8200000°E	*Microtermes pakistanicus*	[27]
		16.4700000°N,102.8200000°E	*Microtermes obesi*	[27]
		16.4700000°N,102.8200000°E	*Odontotermes feae*	[27]
		16.4700000°N,102.8200000°E	*Odontotermes longignathus*	[27]
		16.4700000°N,102.8200000°E	*Odontotermes formosanus*	[27]
		16.4700000°N,102.8200000°E	*Globitermes sulphureus*	[27]
		16.4700000°N,102.8200000°E	*Microcerotermes crassus*	[27]
		16.4700000°N,102.8200000°E	*Coptotermes havilandi*	[27]
Nakhon Ratchasima	Mueang Nakhon Ratchasima	14.9558770°N,102.1784710°E	*Odontotermes proformosanus*	[37]
		14.9558770°N,102.1784710°E	*Odontotermes feae*	[37]
		14.9558770°N,102.1784710°E	*Odontotermes* sp.1	[37]
		14.9558770°N,102.1784710°E	*Macrotermes gilvus*	[37]
		14.9558770°N,102.1784710°E	*Macrotermes chaiglomi*	[37]
		14.9558770°N,102.1784710°E	*Macrotermes annandalei*	[37]
		14.9558770°N,102.1784710°E	*Microtermes obesi*	[37]
		14.9558770°N,102.1784710°E	*Dicuspiditermes* sp.1	[37]
		14.9558770°N,102.1784710°E	*Microcerotermes crassus*	[37]
		14.9558770°N,102.1784710°E	*Globitermes sulphureus*	[37]

Province	District	Geographic Location1/	Termite Species	Citation
		14.9558770°N,102.1784710°E	*Coptotermes gestroi*	[37]
**Eastern region**				
Chanthaburi	Khao Khitchakut	12.8596088°N, 102.1321005°E	*Glyptotermes* sp.1	[28]
		12.8596088°N, 102.1321005°E	*Glyptotermes* sp.2	[28]
		12.8596088°N, 102.1321005°E	*Glyptotermes* sp.3	[28]
		12.8596088°N, 102.1321005°E	*Schedorhinotermes medioobscurus*	[28]
		12.8596088°N, 102.1321005°E	*Coptotermes gestroi*	[28]
		12.8596088°N, 102.1321005°E	*Coptotermes havilandi*	[28]
		12.8596088°N, 102.1321005°E	*Coptotermes premrasmii*	[28]
		12.8596088°N, 102.1321005°E	*Macrotermes annandalei*	[28]
		12.8596088°N, 102.1321005°E	*Macrotermes maesodensis*	[28]
		12.8596088°N, 102.1321005°E	*Microtermes obesi*	[28]
		12.8596088°N, 102.1321005°E	*Ancistrotermes pakestanicus*	[28]
		12.8596088°N, 102.1321005°E	*Hypotermes makhamensis*	[28]
		12.8596088°N, 102.1321005°E	*Odontotermes proformosanus*	[28]
		12.8596088°N, 102.1321005°E	*Odontotermes formosanus*	[28]
		12.8596088°N, 102.1321005°E	*Odontotermes oblongathus*	[28]
		12.8596088°N, 102.1321005°E	*Odontotermes* sp.2	[28]
		12.8596088°N, 102.1321005°E	*Microcerotermes crassus*	[28]
		12.8596088°N, 102.1321005°E	*Microcerotermes distans*	[28]

Province	District	Geographic Location[1/]	Termite Species	Citation
		12.8596088°N, 102.1321005°E	*Microcerotermes annandalei*	[28]
		12.8596088°N, 102.1321005°E	*Globitermes sulphureus*	[28]
		12.8596088°N, 102.1321005°E	*Termes cosmis*	[28]
		12.8596088°N, 102.1321005°E	*Mirocapritermes concaveus*	[28]
		12.8596088°N, 102.1321005°E	*Angulitermes* sp.	[28]
		12.8596088°N, 102.1321005°E	*Euhamitermes* sp.	[28]
		12.8596088°N, 102.1321005°E	*Speculitermes* sp.	[28]
		12.8596088°N, 102.1321005°E	*Nasutitermes johoricus*	[28]
		12.8596088°N, 102.1321005°E	*Nasutitermes* sp.1	[28]
		12.8596088°N, 102.1321005°E	*Nasutitermes* sp.5	[28]
		12.8596088°N, 102.1321005°E	*Bulbitermes parapusillus*	[28]
		12.8596088°N, 102.1321005°E	*Bulbitermes laticephalus*	[28]
		12.8596088°N, 102.1321005°E	*Bulbitermes* sp.2	[28]
		12.8596088°N, 102.1321005°E	*Hospitalitermes ataramensis*	[28]
		12.8596088°N, 102.1321005°E	*Hospitalitermes* sp.3	[28]
		12.8596088°N, 102.1321005°E	*Hospitalitermes* sp.4	[28]
Chanthaburi	Khao Khitchakut	12.9750204°N, 102.1606711°E	*Cryptotermes thailandis*	[28]
		12.9750204°N, 102.1606711°E	*Glyptotermes brevicandatus*	[28]
		12.9750204°N, 102.1606711°E	*Glyptotermes* sp.2	[28]
		12.9750204°N, 102.1606711°E	*Schedorhinotermes medioobscurus*	[28]

Province	District	Geographic Location[1/]	Termite Species	Citation
		12.9750204°N, 102.1606711°E	*Schedorhinotermes rectangularis*	[28]
		12.9750204°N, 102.1606711°E	*Macrotermes annandalei*	[28]
		12.9750204°N, 102.1606711°E	*Ancistrotermes pakestanicus*	[28]
		12.9750204°N, 102.1606711°E	*Hypotermes makhamensis*	[28]
		12.9750204°N, 102.1606711°E	*Odontotermes proformosanus*	[28]
		12.9750204°N, 102.1606711°E	*Odontotermes formosanus*	[28]
		12.9750204°N, 102.1606711°E	*Pericapritermes semarangi*	[28]
		12.9750204°N, 102.1606711°E	*Pericapritermes latignathus*	[28]
		12.9750204°N, 102.1606711°E	*Nasutitermes* sp.1	[28]
		12.9750204°N, 102.1606711°E	*Nasutitermes* sp.4	[28]
		12.9750204°N, 102.1606711°E	*Nasutitermes* sp.5	[28]
		12.9750204°N, 102.1606711°E	*Bulbitermes parapusillus*	[28]
		12.9750204°N, 102.1606711°E	*Bulbitermes laticephalus*	[28]
		12.9750204°N, 102.1606711°E	*Bulbitermes* sp.1	[28]
		12.9750204°N, 102.1606711°E	*Bulbitermes* sp.3	[28]
		12.9750204°N, 102.1606711°E	*Hospitalitermes jepsoni*	[28]
		12.9750204°N, 102.1606711°E	*Hospitalitermes* sp.3	[28]
		12.9750204°N, 102.1606711°E	*Lacessititermes* sp.1	[28]
		12.9750204°N, 102.1606711°E	*Lacessititermes* sp.2	[28]
Chanthaburi	Khao	12.6333330°N, 102.2166670°E	*Macrotermes carbonarius*	[33]

Province	District	Geographic Location[1/]	Termite Species	Citation
	Khitchakut	12.6333330°N, 102.2166670°E	*Microtermes pakistanicus*	[33]
		12.6333330°N, 102.2166670°E	*Microtermes obesi*	[33]
		12.6333330°N, 102.2166670°E	*Odontotermes formosanus*	[33]
		12.6333330°N, 102.2166670°E	*Nasutitermes matangensiformis*	[33]
		12.6333330°N, 102.2166670°E	*Dicuspiditermes makhamensis*	[33]
		12.6333330°N, 102.2166670°E	*Globitermes sulphureus*	[33]
		12.6333330°N, 102.2166670°E	*Microcerotermes crassus*	[33]
		12.6333330°N, 102.2166670°E	*Termes cosmis*	[33]
Chanthaburi	Soi Dao	13.1052313°N, 102.2000951°E	*Cryptotermes thailandis*	[28]
		13.1052313°N, 102.2000951°E	*Glyptotermes* sp.2	[28]
		13.1052313°N, 102.2000951°E	*Schedorhinotermes medioobscurus*	[28]
		13.1052313°N, 102.2000951°E	*Coptotermes gestroi*	[28]
		13.1052313°N, 102.2000951°E	*Coptotermes curvignathus*	[28]
		13.1052313°N, 102.2000951°E	*Coptotermes* sp.1	[28]
		13.1052313°N, 102.2000951°E	*Macrotermes annandalei*	[28]
		13.1052313°N, 102.2000951°E	*Microtermes obesi*	[28]
		13.1052313°N, 102.2000951°E	*Ancistrotermes pakestanicus*	[28]
		13.1052313°N, 102.2000951°E	*Hypotermes makhamensis*	[28]
		13.1052313°N, 102.2000951°E	*Odontotermes proformosanus*	[28]
		13.1052313°N, 102.2000951°E	*Odontotermes formosanus*	[28]

Province	District	Geographic Location1/	Termite Species	Citation
		13.1052313°N, 102.2000951°E	*Microcerotermes crassus*	[28]
		13.1052313°N, 102.2000951°E	*Microcerotermes distans*	[28]
		13.1052313°N, 102.2000951°E	*Globitermes sulphureus*	[28]
		13.1052313°N, 102.2000951°E	*Termes cosmis*	[28]
		13.1052313°N, 102.2000951°E	*Pericapritermes* sp.C	[28]
		13.1052313°N, 102.2000951°E	*Nasutitermes johoricus*	[28]
		13.1052313°N, 102.2000951°E	*Nasutitermes matangensiformis*	[28]
		13.1052313°N, 102.2000951°E	*Nasutitermes* sp.1	[28]
		13.1052313°N, 102.2000951°E	*Nasutitermes* sp.2	[28]
		13.1052313°N, 102.2000951°E	*Nasutitermes* sp.3	[28]
		13.1052313°N, 102.2000951°E	*Nasutitermes* sp.4	[28]
		13.1052313°N, 102.2000951°E	*Nasutitermes* sp.5	[28]
		13.1052313°N, 102.2000951°E	*Bulbitermes parapusillus*	[28]
		13.1052313°N, 102.2000951°E	*Bulbitermes laticephalus*	[28]
		13.1052313°N, 102.2000951°E	*Bulbitermes* sp.2	[28]
**Western region**				
Kanchanaburi	Si Sawat	14.6272859°N, 98.9994688°E	*Glyptotermes pinangae*	[31]
		14.6272859°N, 98.9994688°E	*Glyptotermes brevicaudatus*	[31]
		14.6272859°N, 98.9994688°E	*Glyptotermes* sp.2	[31]
		14.6272859°N, 98.9994688°E	*Schedorhinotermes medioobscurus*	[31]

Province	District	Geographic Location[1/]	Termite Species	Citation
		14.6272859°N, 98.9994688°E	*Coptotermes havilandi*	[31]
		14.6272859°N, 98.9994688°E	*Microtermes obesi*	[31]
		14.6272859°N, 98.9994688°E	*Ancistrotermes pakestanicus*	[31]
		14.6272859°N, 98.9994688°E	*Macrotermes gilvus*	[31]
		14.6272859°N, 98.9994688°E	*Macrotermes chaiglomi*	[31]
		14.6272859°N, 98.9994688°E	*Macrotermes annandalei*	[31]
		14.6272859°N, 98.9994688°E	*Macrotermes maesodensis*	[31]
		14.6272859°N, 98.9994688°E	*Odontotermes proformosanus*	[31]
		14.6272859°N, 98.9994688°E	*Odontotermes formosanus*	[31]
		14.6272859°N, 98.9994688°E	*Odontotermes longignathus*	[31]
		14.6272859°N, 98.9994688°E	*Odontotermes feae*	[31]
		14.6272859°N, 98.9994688°E	*Odontotermes takensis*	[31]
		14.6272859°N, 98.9994688°E	*Odontotermes maesodensis*	[31]
		14.6272859°N, 98.9994688°E	*Odontotermes* sp.2	[31]
		14.6272859°N, 98.9994688°E	*Odontotermes* sp.3	[31]
		14.6272859°N, 98.9994688°E	*Odontotermes* sp.5	[31]
		14.6272859°N, 98.9994688°E	*Odontotermes* sp.8	[31]
		14.6272859°N, 98.9994688°E	*Odontotermes* sp.10	[31]
		14.6272859°N, 98.9994688°E	*Odontotermes* sp.11	[31]
		14.6272859°N, 98.9994688°E	*Odontotermes* sp.12	[31]

Province	District	Geographic Location[1/]	Termite Species	Citation
		14.6272859°N, 98.9994688°E	*Odontotermes* sp.13	[31]
		14.6272859°N, 98.9994688°E	*Odontotermes* sp.16	[31]
		14.6272859°N, 98.9994688°E	*Odontotermes* sp.17 *(- sarawakensis)*	[31]
		14.6272859°N, 98.9994688°E	*Globitermes sulphureus*	[31]
		14.6272859°N, 98.9994688°E	*Microcerotermes annandalei*	[31]
		14.6272859°N, 98.9994688°E	*Microcerotermes minutus*	[31]
		14.6272859°N, 98.9994688°E	*Microcerotermes* sp.1	[31]
		14.6272859°N, 98.9994688°E	*Dicuspiditermes garthwaitei*	[31]
		14.6272859°N, 98.9994688°E	*Pericapritermes* sp.E	[31]
		14.6272859°N, 98.9994688°E	*Pericapritermes* sp.F	[31]
		14.6272859°N, 98.9994688°E	*Pericapritermes* sp.G	[31]
		14.6272859°N, 98.9994688°E	*Pericapritermes latignathus*	[31]
		14.6272859°N, 98.9994688°E	*Euhamitermes hamatus*	[31]
		14.6272859°N, 98.9994688°E	*Speculitermes macrodentatus*	[31]
		14.6272859°N, 98.9994688°E	*Speculitermes* sp.	[31]
		14.6272859°N, 98.9994688°E	*Nasutitermes dimorphus*	[31]
		14.6272859°N, 98.9994688°E	*Nasutitermes perparvus*	[31]
		14.6272859°N, 98.9994688°E	*Nasutitermes matangensiformis*	[31]
		14.6272859°N, 98.9994688°E	*Bulbitermes prabhae*	[31]
		14.6272859°N, 98.9994688°E	*Bulbitermes* sp.	[31]

Province	District	Geographic Location[1/]	Termite Species	Citation
		14.6272859°N, 98.9994688°E	*Bulbitermes* sp.4	[31]
		14.6272859°N, 98.9994688°E	*Bulbitermes parapusillus*	[31]
		14.6272859°N, 98.9994688°E	*Havilanditermes* sp.	[31]
Kanchanaburi	Si Sawat	14.6148305°N, 98.9912486°E	*Coptotermes havilandi*	[28]
		14.6148305°N, 98.9912486°E	*Macrotermes gilvus*	[28]
		14.6148305°N, 98.9912486°E	*Macrotermes chaiglomi*	[28]
		14.6148305°N, 98.9912486°E	*Microtermes obesi*	[28]
		14.6148305°N, 98.9912486°E	*Ancistrotermes pakestanicus*	[28]
		14.6148305°N, 98.9912486°E	*Odontotermes proformosanus*	[28]
		14.6148305°N, 98.9912486°E	*Odontotermes formosanus*	[28]
		14.6148305°N, 98.9912486°E	*Odontotermes longignathus*	[28]
		14.6148305°N, 98.9912486°E	*Odontotermes feae*	[28]
		14.6148305°N, 98.9912486°E	*Odontotermes takensis*	[28]
		14.6148305°N, 98.9912486°E	*Odontotermes maesodensis*	[28]
		14.6148305°N, 98.9912486°E	*Odontotermes* sp.2	[28]
		14.6148305°N, 98.9912486°E	*Odontotermes* sp.3	[28]
		14.6148305°N, 98.9912486°E	*Odontotermes* sp.5	[28]
		14.6148305°N, 98.9912486°E	*Odontotermes* sp.8	[28]
		14.6148305°N, 98.9912486°E	*Odontotermes* sp.10	[28]
		14.6148305°N, 98.9912486°E	*Odontotermes* sp.11	[28]

Province	District	Geographic Location[1/]	Termite Species	Citation
		14.6148305°N, 98.9912486°E	*Odontotermes* sp.12	[28]
		14.6148305°N, 98.9912486°E	*Odontotermes* sp.13	[28]
		14.6148305°N, 98.9912486°E	*Odontotermes* sp.16	[28]
		14.6148305°N, 98.9912486°E	*Microcerotermes annandalei*	[28]
		14.6148305°N, 98.9912486°E	*Microcerotermes minutus*	[28]
		14.6148305°N, 98.9912486°E	*Microcerotermes* sp.1	[28]
		14.6148305°N, 98.9912486°E	*Globitermes sulphureus*	[28]
		14.6148305°N, 98.9912486°E	*Dicuspiditermes garthwaitei*	[28]
		14.6148305°N, 98.9912486°E	*Pericapritermes latignathus*	[28]
		14.6148305°N, 98.9912486°E	*Pericapritermes* sp.E	[28]
		14.6148305°N, 98.9912486°E	*Pericapritermes* sp.F	[28]
		14.6148305°N, 98.9912486°E	*Pericapritermes* sp.G	[28]
		14.6148305°N, 98.9912486°E	*Euhamitermes hamatus*	[28]
		14.6148305°N, 98.9912486°E	*Speculitermes macrodentatus*	[28]
		14.6148305°N, 98.9912486°E	*Nasutitermes dimorphus*	[28]
		14.6148305°N, 98.9912486°E	*Nasutitermes perparvus*	[28]
		14.6148305°N, 98.9912486°E	*Bulbitermes prabhae*	[28]
		14.6148305°N, 98.9912486°E	*Bulbitermes* sp.	[28]
**Southern region**				
Phang Nga	Khura	9.0594292°N, 98.2695751°E	*Neotermes* sp.2	[29]

Province	District	Geographic Location1/	Termite Species	Citation
	Buri	9.0594292°N, 98.2695751°E	*Schedorhinotermes rectangularis*	[29]
		9.0594292°N, 98.2695751°E	*Schedorhinotermes medioobscurus*	[29]
		9.0594292°N, 98.2695751°E	*Schedorhinotermes sarawakensis*	[29]
		9.0594292°N, 98.2695751°E	*Coptotermes havilandi*	[29]
		9.0594292°N, 98.2695751°E	*Odontotermes proformosanus*	[29]
		9.0594292°N, 98.2695751°E	*Ancistrotermes pakistanicus*	[29]
		9.0594292°N, 98.2695751°E	*Amitermes dentatus*	[29]
		9.0594292°N, 98.2695751°E	*Globitermes sulphureus*	[29]
		9.0594292°N, 98.2695751°E	*Microcerotermes crassus*	[29]
		9.0594292°N, 98.2695751°E	*Microcerotermes* sp.	[29]
		9.0594292°N, 98.2695751°E	*Termes propinquus*	[29]
		9.0594292°N, 98.2695751°E	*Pericapritermes semarangi*	[29]
		9.0594292°N, 98.2695751°E	*Pericapritermes* sp.B	[29]
		9.0594292°N, 98.2695751°E	*Pericapritermes* sp.E	[29]
		9.0594292°N, 98.2695751°E	*Pericapritermes* sp.F	[29]
		9.0594292°N, 98.2695751°E	*Procapritermes longignathus*	[29]
		9.0594292°N, 98.2695751°E	*Procapritermes prosetiger*	[29]
		9.0594292°N, 98.2695751°E	*Procapritermes parasilvaticus*	[29]
		9.0594292°N, 98.2695751°E	*Bulbitermes parapusillus*	[29]
		9.0594292°N, 98.2695751°E	*Bulbitermes prabhae*	[29]

Province	District	Geographic Location[1/]	Termite Species	Citation
		9.0594292°N, 98.2695751°E	*Nasutitermes matangensiformis*	[29]
		9.0594292°N, 98.2695751°E	*Nasutitermes tungsalangensis*	[29]
		9.0594292°N, 98.2695751°E	*Hospitalitermes ataramensis*	[29]
		9.0594292°N, 98.2695751°E	*Havilanditermes proatripennis*	[29]
Phang Nga	Khura Buri	9.1806991°N, 98.2806151°E	*Cryptotermes thailandis*	[29]
		9.1806991°N, 98.2806151°E	*Glyptotermes* sp.	[29]
		9.1806991°N, 98.2806151°E	*Schedorhinotermes medioobscurus*	[29]
		9.1806991°N, 98.2806151°E	*Schedorhinotermes sarawakensis*	[29]
		9.1806991°N, 98.2806151°E	*Prorhinotermes tibiaoensiformis*	[29]
		9.1806991°N, 98.2806151°E	*Coptotermes havilandi*	[29]
		9.1806991°N, 98.2806151°E	*Coptotermes premrasmii*	[29]
		9.1806991°N, 98.2806151°E	*Macrotermes gilvus*	[29]
		9.1806991°N, 98.2806151°E	*Odontotermes proformosanus*	[29]
		9.1806991°N, 98.2806151°E	*Odontotermes formosanus*	[29]
		9.1806991°N, 98.2806151°E	*Odontotermes longignathus*	[29]
		9.1806991°N, 98.2806151°E	*Odontotermes feae*	[29]
		9.1806991°N, 98.2806151°E	*Odontotermes maesodensis*	[29]
		9.1806991°N, 98.2806151°E	*Odontotermes* sp.1	[29]
		9.1806991°N, 98.2806151°E	*Odontotermes* sp.5	[29]
		9.1806991°N, 98.2806151°E	*Odontotermes* sp.12	[29]

Province	District	Geographic Location[1/]	Termite Species	Citation
		9.1806991°N, 98.2806151°E	*Microtermes obesi*	[29]
		9.1806991°N, 98.2806151°E	*Ancistrotermes pakistanicus*	[29]
		9.1806991°N, 98.2806151°E	*Hypotermes makhamensis*	[29]
		9.1806991°N, 98.2806151°E	*Amitermes dentatus*	[29]
		9.1806991°N, 98.2806151°E	*Globitermes sulphureus*	[29]
		9.1806991°N, 98.2806151°E	*Microcerotermes crassus*	[29]
		9.1806991°N, 98.2806151°E	*Microcerotermes paracelebensis*	[29]
		9.1806991°N, 98.2806151°E	*Termes propinquus*	[29]
		9.1806991°N, 98.2806151°E	*Pericapritermes* sp.E	[29]
		9.1806991°N, 98.2806151°E	*Nasutitermes matangensiformis*	[29]
		9.1806991°N, 98.2806151°E	*Nasutitermes tungsalangensis*	[29]
		9.1806991°N, 98.2806151°E	*Nasutitermes* sp. (<tung)	[29]
		9.1806991°N, 98.2806151°E	*Nasutitermes* sp.2	[29]
Phang Nga	Thai Mueang	8.4802776°N, 97.6466667°E	*Cryptotermes* sp.	[34]
		8.4802776°N, 97.6466667°E	*Schedorhinotermes sarawakensis*	[34]
		8.4802776°N, 97.6466667°E	*Macrotermes maesodensis*	[34]
		8.4802776°N, 97.6466667°E	*Hypotermes makhamensis*	[34]
		8.4802776°N, 97.6466667°E	*Odontotermes proformosanus*	[34]
		8.4802776°N, 97.6466667°E	*Odontotermes* sp.	[34]
		8.4802776°N, 97.6466667°E	*Microcerotermes crassus*	[34]

Province	District	Geographic Location1/	Termite Species	Citation
		8.4802776°N, 97.6466667°E	*Microcerotermes paracelebensis*	[34]
		8.4802776°N, 97.6466667°E	*Globitermes sulphureus*	[34]
		8.4802776°N, 97.6466667°E	*Nasutitermes matangensiformis*	[34]
		8.4802776°N, 97.6466667°E	*Nasutitermes johoricus*	[34]
		8.4802776°N, 97.6466667°E	*Nasutitermes* sp.	[34]
Phang Nga	Thai Mueang	8.5005851°N, 97.6426162°	*Cryptotermes* sp.	[34]
		8.5005851°N, 97.6426162°	*Neotermes* sp.	[34]
		8.5005851°N, 97.6426162°	*Macrotermes maesodensis*	[34]
		8.5005851°N, 97.6426162°	*Hypotermes makhamensis*	[34]
		8.5005851°N, 97.6426162°	*Odontotermes proformosanus*	[34]
		8.5005851°N, 97.6426162°	*Microcerotermes crassus*	[34]
		8.5005851°N, 97.6426162°	*Microcerotermes paracelebensis*	[34]
		8.5005851°N, 97.6426162°	*Globitermes sulphureus*	[34]
		8.5005851°N, 97.6426162°	*Dicuspiditermes garthwaitei*	[34]
		8.5005851°N, 97.6426162°	*Pericapritermes* sp.	[34]
		8.5005851°N, 97.6426162°	*Nasutitermes matangensiformis*	[34]
		8.5005851°N, 97.6426162°	*Nasutitermes johoricus*	[34]
		8.5005851°N, 97.6426162°	*Nasutitermes* sp.	[34]
Phang Nga	Thai Mueang	8.5679177°N, 97.6359631°E	*Neotermes* sp.	[34]
		8.5679177°N, 97.6359631°E	*Schedorhinotermes sarawakensis*	[34]

Province	District	Geographic Location[1/]	Termite Species	Citation
		8.5679177°N, 97.6359631°E	*Macrotermes maesodensis*	[34]
		8.5679177°N, 97.6359631°E	*Odontotermes proformosanus*	[34]
		8.5679177°N, 97.6359631°E	*Odontotermes* sp.	[34]
		8.5679177°N, 97.6359631°E	*Microcerotermes crassus*	[34]
		8.5679177°N, 97.6359631°E	*Microcerotermes paracelebensis*	[34]
		8.5679177°N, 97.6359631°E	*Globitermes sulphureus*	[34]
		8.5679177°N, 97.6359631°E	*Dicuspiditermes garthwaitei*	[34]
		8.5679177°N, 97.6359631°E	*Pericapritermes* sp.	[34]
		8.5679177°N, 97.6359631°E	*Nasutitermes johoricus*	[34]
Phang Nga	Thai Mueang	8.5914870°N, 97.6379430°E	*Cryptotermes* sp.	[34]
		8.5914870°N, 97.6379430°E	*Neotermes* sp.	[34]
		8.5914870°N, 97.6379430°E	*Schedorhinotermes sarawakensis*	[34]
		8.5914870°N, 97.6379430°E	*Macrotermes maesodensis*	[34]
		8.5914870°N, 97.6379430°E	*Odontotermes proformosanus*	[34]
		8.5914870°N, 97.6379430°E	*Odontotermes* sp.	[34]
		8.5914870°N, 97.6379430°E	*Microcerotermes crassu*	[34]
		8.5914870°N, 97.6379430°E	*Globitermes sulphureus*	[34]
		8.5914870°N, 97.6379430°E	*Pericapritermes* sp.	[34]
		8.5914870°N, 97.6379430°E	*Nasutitermes matangensiformis*	[34]
		8.5914870°N, 97.6379430°E	*Nasutitermes johoricus*	[34]

Province	District	Geographic Location[1/]	Termite Species	Citation
		8.5914870°N, 97.6379430°E	*Hospitalitermes ataramensis*	[34]
Phang Nga	Thai Mueang	8.6487350°N, 97.6452604°E	*Cryptotermes* sp.	[34]
		8.6487350°N, 97.6452604°E	*Neotermes* sp.	[34]
		8.6487350°N, 97.6452604°E	*Schedorhinotermes sarawakensis*	[34]
		8.6487350°N, 97.6452604°E	*Schedorhinotermes* sp.	[34]
		8.6487350°N, 97.6452604°E	*Macrotermes maesodensis*	[34]
		8.6487350°N, 97.6452604°E	*Odontotermes proformosanus*	[34]
		8.6487350°N, 97.6452604°E	*Odontotermes* sp.	[34]
		8.6487350°N, 97.6452604°E	*Microcerotermes crassus*	[34]
		8.6487350°N, 97.6452604°E	*Globitermes sulphureus*	[34]
		8.6487350°N, 97.6452604°E	*Dicuspiditermes garthwaitei*	[34]
		8.6487350°N, 97.6452604°E	*Pericapritermes* sp.	[34]
		8.6487350°N, 97.6452604°E	*Procapritermes* sp.	[34]
		8.6487350°N, 97.6452604°E	*Mirocapritermes* sp.	[34]
		8.6487350°N, 97.6452604°E	*Homallotermes* sp.	[34]
		8.6487350°N, 97.6452604°E	*Nasutitermes matangensiformis*	[34]
		8.6487350°N, 97.6452604°E	*Nasutitermes johoricus*	[34]
		8.6487350°N, 97.6452604°E	*Hospitalitermes ataramensis*	[34]
		8.6487350°N, 97.6452604°E	*Bulbitermes laticephalus*	[34]
Phang Nga	Thai	8.6788887°N, 97.6483333°E	*Cryptotermes sp.*	[34]

Province	District	Geographic Location[1/]	Termite Species	Citation
	Mueang	8.6788887°N, 97.6483333°E	*Schedorhinotermes sarawakensis*	[34]
		8.6788887°N, 97.6483333°E	*Macrotermes maesodensis*	[34]
		8.6788887°N, 97.6483333°E	*Odontotermes proformosanus*	[34]
		8.6788887°N, 97.6483333°E	*Odontotermes* sp.	[34]
		8.6788887°N, 97.6483333°E	*Microcerotermes crassus*	[34]
		8.6788887°N, 97.6483333°E	*Microcerotermes paracelebensis*	[34]
		8.6788887°N, 97.6483333°E	*Globitermes sulphureus*	[34]
		8.6788887°N, 97.6483333°E	*Pericapritermes* sp.	[34]
		8.6788887°N, 97.6483333°E	*Nasutitermes matangensiformis*	[34]
		8.6788887°N, 97.6483333°E	*Nasutitermes johoricus*	[34]
Chumphon	Pathio	10.8479002°N, 99.4809390°E	*Cryptotermes thailandis*	[32]
		10.8479002°N, 99.4809390°E	*Neotermes* sp.2	[32]
		10.8479002°N, 99.4809390°E	*Coptotermes havilandi*	[32]
		10.8479002°N, 99.4809390°E	*Schedorhinotermes sarawakensis*	[32]
		10.8479002°N, 99.4809390°E	*Macrotermes gilvus*	[32]
		10.8479002°N, 99.4809390°E	*Macrotermes annandalei*	[32]
		10.8479002°N, 99.4809390°E	*Odontotermes proformosanus*	[32]
		10.8479002°N, 99.4809390°E	*Hypotermes makhamensis*	[32]
		10.8479002°N, 99.4809390°E	*Microtermes obesi*	[32]
		10.8479002°N, 99.4809390°E	*Microcerotermes crassus*	[32]

Province	District	Geographic Location[1/]	Termite Species	Citation
		10.8479002°N, 99.4809390°E	*Microcerotermes minutus*	[32]
		10.8479002°N, 99.4809390°E	*Globitermes sulphureus*	[32]
		10.8479002°N, 99.4809390°E	*Pericapritermes* sp.E	[32]
		10.8479002°N, 99.4809390°E	*Pericapritermes* sp.F	[32]
		10.8479002°N, 99.4809390°E	*Nasutitermes johoricus*	[32]
		10.8479002°N, 99.4809390°E	*Nasutitermes matangensiformis*	[32]
		10.8479002°N, 99.4809390°E	*Nasutitermes* sp.	[32]
		10.8479002°N, 99.4809390°E	*Hospitalitermes* sp.1	[32]
Chumphon	Pathio	10.7013887°N, 99.4100000°E	*Cryptotermes thailandis*	[32]
		10.7013887°N, 99.4100000°E	*Odontotermes proformosanus*	[32]
		10.7013887°N, 99.4100000°E	*Microcerotermes crassus*	[32]
		10.7013887°N, 99.4100000°E	*Microcerotermes minutus*	[32]
		10.7013887°N, 99.4100000°E	*Globitermes sulphureus*	[32]
		10.7013887°N, 99.4100000°E	*Pericapritermes* sp.F	[32]

[1/] Geographic Location (Latitude and Longitude) was recorded by Google Earth version 9.121.0.5 and Google Maps application version 10.61.2.

[2/] Unknown species in each subfamily and family
